# Effect of chemotherapy counseling by pharmacists on quality of life and psychological outcomes of oncology patients in Malaysia: a randomized control trial

**DOI:** 10.1186/s12955-017-0680-2

**Published:** 2017-05-15

**Authors:** Ummavathy Periasamy, Sherina Mohd Sidik, Lekhraj Rampal, Siti Irma Fadhilah, Mehrnoosh Akhtari-Zavare, Rozi Mahmud

**Affiliations:** 1 Hospital Tuanku Jaafar, Seremban, Negeri Symbian Malaysia; 2 Cancer Resource & Education Center, Universiti Putra Malaysia, 43400 Serdang, Selangor Malaysia; 3 Department of Psychiatry, Faculty of Medicine & Health Sciences, Universiti Putra Malaysia, 43400 Serdang, Selangor Malaysia; 4 Department of Community Health, Faculty of Medicine & Health Sciences, Universiti Putra Malaysia, 43400 Serdang, Selangor Malaysia; 5 Department of Public Health, Tehran Medical Sciences Branch, Islamic Azad University, Tehran, Iran

**Keywords:** Cancer, Chemotherapy, Quality of life, Psychological outcomes, Counseling, Pharmacist, Malaysia

## Abstract

**Background:**

Cancer is now becoming a leading cause of death. Chemotherapy is an important treatment for cancer patients. These patients also need consultation during their treatment to improve quality of life and decrease psychological disorders. The objectives of the study were to develop, implement and evaluate the effectiveness of a chemotherapy counseling module by pharmacists among oncology patients on their quality of life and psychological outcomes in Malaysia.

**Method:**

A single-blind randomized controlled trial was carried out among 162 oncology patients undergoing chemotherapy from July 2013 to February 2014 in a government hospital with oncology facilities in Malaysia. Participants were randomized to either the intervention group or the control group. Chemotherapy counseling using the module on ‘Managing Patients on Chemotherapy’ by Pharmacists was delivered to the intervention group. The outcome measures were assessed at baseline, first follow-up and second follow-up and third follow-up post-intervention. Chi-square, independent samples t-test and two-way repeated measures ANOVA were conducted in the course of the data analyses.

**Results:**

In assessing the impact of the chemotherapy counseling module, the study revealed that the module along with repetitive counseling showed significant improvement of quality of life in the intervention group as compared to the control group with a large effect size in physical health (*p* = 0.001, partial Ƞ^2^ = 0.66), psychological (*p* = 0.001, partial Ƞ^2^ = 0.65), social relationships (*p* = 0.001, partial Ƞ^2^ = 0.30), and environment (*p* = 0.001, partial Ƞ^2^ = 0.67) and decrease in the anxiety (*p* = 0.000; partial Ƞ^2^ = 0.23), depression (*p* = 0.000; partial Ƞ^2^ = 0.40).

**Conclusion:**

The module on ‘Managing Patients on Chemotherapy’ along with repetitive counseling by pharmacists has been shown to be effective in improving quality of life and decreasing anxiety and depression among oncology patients undergoing chemotherapy.

**Trial registration number:**

National Medical Research Register (NMRR) of Malaysia and given a registration number NMRR-12-1057-12,363 on 21 December 2012.

## Background

Cancer is the second leading cause of death globally and accounted for 8.8 million death (approximately 16% of total deaths) in 2015 [1]. At least 30–50% of all cancers can be prevented by healthy lifestyle choices such as avoidance of smoking and tobacco exposure, drinking alcohol, inadequate exercise or being overweight [1]. In Malaysia, cancer is one of the leading causes of death [2]. Based on the National Cancer Registry [3] a total of 103,507 new cancer cases were diagnosed in Malaysia for the period of 2007–2011 which increased five times from 2003 (21,464 cases of cancer) [3]. Although, the number of new cases in Malaysia increased but the survival rate of cancer is also increasing. Therefore, improving quality of life and psychological outcomes among cancer survivors in Malaysia and the world would have significant public health implications [2, 4].

Chemotherapy and radiotherapy are the most common types of treatment for cancer patients which have contributed to the increase of their survival rates [5, 6]. Globally, these treatments are known to have damaging psychological effects including depression, anxiety and poor quality of life for cancer patients [7]. A study by the World Health Organization found that chemotherapy affects the health related quality of life (HRQOL) positively by alleviating symptoms, halting or reversing deteriorations, but has negative impact of side effects. As chemotherapy is systemic, side effects are similar regardless of cancer types [8].

Quality of life (QOL) is a multidimensional, multifaceted measure which refers to a patient’s perception of general wellbeing, including psychological, cognitive, physical and social functioning [9]. Recently, QOL has been viewed as a primary end point to assess the quality of care and management in oncology medicine [10]. Numerous studies conducted in Malaysia [11], China [4] and Scotland [12], showed that the quality of life of cancer patients is an important predictor of survival, and psychological problems of cancer patients should be considered by physicians before treatment of cancer patients.

Anxiety and depression are another side effects of chemotherapy which are the common psychological disorders among cancer patients and difficult to be detected and treated [6]. Cancer patients suffering these symptoms for a long time after the end of chemotherapy and also manifested in the recurrence of the disease [13]. Consequently, it is important for healthcare professionals to identify signs of anxiety and depression among cancer patients [14].

Therefore, an increasingly important issue in oncology is to evaluate QOL and psychological issue in cancer patients [15]. The cancer-specific QOL and psychological issues are related to all stages of the disease. It is important to know that, most of the cancer patients suffer from side effects of chemotherapy which observed by pharmacists who are in charge of administering chemotherapy to cancer patients. Consequently, pharmacists must have a role in helping cancer patients to overcome or cope with chemotherapy treatment side effects. Currently, in Malaysia there was not any module/guild line for pharmacists and they usually relied on their own knowledge and experience addressing problems faced by cancer patients during chemotherapy. The aim of this study was to develop, implement and evaluate the effect of a chemotherapy counseling module by pharmacists among oncology patients based on their QOL and Psychological issue.

## Methods

### Study design

A single-blind randomized control trial study was conducted between July 1th, 2013 and February 28th 2014 in selected government hospital in Malaysia**.**

### Recruitment and randomization

All Malaysia patients above 18 years old in different stages of cancers which undergoing their 1th and 2nd cycles of chemotherapy and able to read were recruited. Those who had already undergone their third cycle of chemotherapy onwards, very sick and had speech and communication disorders were excluded from the study.

The list of all cancer patients who met the inclusion criteria was obtained from the cytotoxic drug reconstitution (CDR) of the Pharmacy Department of the hospital and served as a sampling frame for this study. The eligible patients were randomly assigned into the intervention and control groups from the sampling frame by using the even and odd numbers selection. The even numbers were assigned to the control group, while the odd numbers were assigned to the intervention group. Patient recruitment occurred on a daily basis and was based on the number of registered patients. It was continue until a total of 162 patients were obtained for both intervention and control groups. In order to maintain confidentiality the unique code numbers were given to each member of both the control and intervention groups and used by them in the questionnaire.

### Blinding

Blinding of the participants or the investigators to the allocation groups was not possible, however, the data entry and analysis were carried out by an independent team led by a statistician.

### Development of intervention

The module on ‘Managing Patients on Chemotherapy’ by Pharmacists was developed through focus group discussion (FGD) and pilot test among forty cancer patients undergoing chemotherapy which not included in the main study. The FGD was conducted based on the set of questions which are mention in Table 1. Feedback from patients during the FGD and pilot test was noted, and combined with the “Chemotherapy and You” module by the National Cancer Institute (NCI) [8, 16]. Then, the new module “Managing Patients on Chemotherapy” module was checked by a group of experts including Family Medicine, Pharmacy, Oncology, Public Health, Nutrition, and Psychology. The module covers a wide range of areas, which include information on chemotherapy, side effects of chemo drugs and how to manage common chemotherapy side-effects, how to prepare for chemotherapy (before, during and after chemotherapy), what type of food eat or not eat before, during and after chemotherapy, information on psychological aspects of patients undergoing chemotherapy; namely depression, anxiety. This new module provides evidence-based information to pharmacists in counseling patients and emphasizes the importance in spending quality time with the patients as they undergo each chemotherapy cycle. This is not being practiced currently as most pharmacists do not have any guide to refer to, and have to rely on their own knowledge and experience based on each patient’s questions and needs.

**Table 1:** Topic guide for focus group discussion

No	List of questions
1	What would you like to know during chemotherapy?
2	What are the major problems faced before, during and after chemotherapy?
3	How do you want us pharmacists to guide or help you during chemotherapy?
4	How will our support and guidance be helpful?
5	What are the information needed during counselling after chemotherapy?

### Intervention

The intervention group received chemotherapy counseling by the pharmacist-in-charge of this study based on the “Managing Patients on Chemotherapy’ module during their baseline, 1st follow-up, 2nd follow-up and 3rd follow-up sessions. The intervention was takes place at designated locations in the hospital and each session is designed to last 45 min ±10 min. The psychological assessment and chemotherapy counseling was done by the pharmacist-in-charge for this study which was trained by a clinical psychologist before starting the actual study.

The patients in the control group received treatment-as-usual (TAU). This consisted of some general explanation by the pharmacist based on their own knowledge and this usually only occurs during the first cycle of chemotherapy. However, the control group received the educational module at the end of study.

All participants in the intervention and control groups responded to a validated and pretested questionnaire at baseline prior to the implementation of the chemotherapy counseling module in the intervention group and usual consultation for control group.

Patients were followed up and assessed during each of their chemotherapy cycle; which varied from 3 to 6 weeks depending on the type of chemotherapy treatment they received. The three follow ups were achieved within 3 to 5 months (12–18 weeks). Patients were recruited from July 2013 to February 2014.

**Fig. 1:**
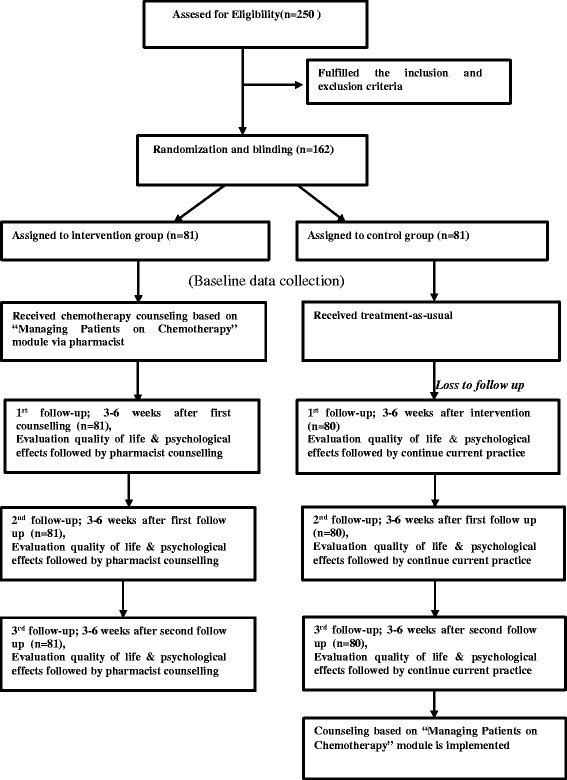
CONSORT diagram of study participants in control and intervention groups

Patients completed their questionnaire during each chemotherapy treatment before the chemotherapy counselling session. This process was repeated in each of the three sequential chemotherapy cycles; which were defined as 1st, 2nd and 3rd follow-up sessions in this study. However, each patient completed their questionnaire at different times according to their chemotherapy treatment. Figure 1 provides the participant CONSORT diagram of both the intervention and control groups.

### Instrument

All questionnaires were administered in Malay using validated translated versions of the original instruments [17–19].

*Socio-demographic characteristics*: Items on the socio-demographic characteristics included age, gender, race, religion, marital status, education level, family history with cancer and stage of cancer. Demographic items were included in baseline questionnaire.

#### WHO quality of life-BREF (WHOQOL-BREF)

The validated Malay version of (WHOQOL-BREF) was used in the study [17]. The WHOQOL-BREF instrument comprises 26 items, which measure the QOL of each patient according to four domains included; physical health (7 items), psychological health (6 items), social relationships (3 items) and environment (8 items). Response categories were in a Likert format [1–5, “very poor” (1) to “very good” (5)], with scores ranging between 26 to 130. There are no cut–off points for any domains. The higher scores represent greater QOL for that particular domain.

#### Patient health questionnaire-9 (PHQ-9)

The PHQ-9 questionnaire was used in this study to determine the depression level encountered by each cancer patient [20]. It consists of 9 items, each item scored as follows; Not at all (0), Several days (1), More than half the days (2) and Nearly every day (3), with an overall range score of 0–27. The validated Malay version of (PHQ-9) which was found to have good sensitivity and specificity was used to determine depression in this study [18].

#### Generalized anxiety disorder-7 (GAD-7)

The Generalized anxiety disorder-7 (GAD-7) questionnaire was used to determine the anxiety level encountered by each cancer patient. The GAD-7 consisted of seven items measuring GAD, post-traumatic stress disorder (PTSD), panic disorder, and social anxiety. Each of the seven items was scored from 0 (not at all) to 3 (nearly every day). Scores of GAD-7 ranged from 0 to 21; where scores of 5, 10, and 15 represent mild, moderate and severe anxiety symptoms, respectively [21]. The validated Malay version of GAD-7 which was found to have good sensitivity and specificity was used in this study [19].

### Sample size estimation

The Rosner’ formula (*n* = [zα√pq(1 + 1/k) + zβ√p1q1 + p2q2/k]2/Δ2) was used for sample size estimation [22]. In order to achieve 80% power (2-sided alpha *P* = 0.05) to detect a group differences 69% [23] with 10% attrition rate, 81 cancer patients in each arm were required.

### Ethics statement

The study protocol was approved by Ethics Committee of faculty of medicine & health science, Universiti Putra Malaysia (Ref No: UPM/TNCPI/RMC/1.4.18.1 (JKEUPM)/F1), as well as director of the selected hospital. This study was registered with the National Medical Research Register (NMRR) of Malaysia and given a registration number NMRR-12-1057-12,363. A written informed consent was taken before performing the study.

### Statistical analysis

Analyses were conducted with statistical computer software of SPSS version 20 (IBM SPSS Statistics 20). The outcomes of interest were quality of life and each domain included (physical health, psychological health, social relationships and environment). Frequency, percentage, mean and standard deviation were used to describe the socio-demographic characteristics of the intervention and control groups. Normality of data was examined by using the Kolmogorov-Smirnov test and all data were normally distributed (*p* > 0.05). At baseline, the independent samples t-test and Chi-square were used to comparison between the control and intervention groups. Two- way repeated measures ANOVA test was employed to look at the main and interaction effects within and between groups for mean scores of QOL (physical health, psychological health, social relationships and environment) and mean scores of psychological effect (anxiety and depression). It used partial eta squared (η^2^) as a measure of effect size which represents the variance proportion in the dependent variable (depression, anxiety, quality of life) that can be explained by the independent variable (received intervention or not). The interpretation of the strength of eta squared values used the guidelines by small effect (0.01), moderate effect (0.06), and large effect (0.14) [24]. Confidence interval was set at 95% for the estimation of odds ratio and mean. Post hoc analysis was used to find where the significant differences actually occurred in the group time at new *p*-value of ≤0.005 after Bonferroni adjustment. A significance level of *p* ≤ 0.05 was used in all analyses.

## Results

### Baseline data

The cancer patients (*n* = 161) who participated in this study were assigned to the intervention (*n* = 81) and control (*n* = 80) groups. The majorities of participants were Malay 84(52.2%), Muslim 84(52.2%) and married 116(72.0%). With regards to educational level, 88(54.7%) of them were diploma & less than diploma and 40(24.8%) were illiterate. The average age of the respondents was 65 years (mean = 65.49 ± 1.4; 95% CI = 64.08–66.90) and majority of them 87(54.0%) don’t have family history of cancer. At baseline, no significant difference was found between the study groups regarding participant characteristics (*p* > 0.05) (Table 2).

**Table 2:** Socio- demographic characteristics of respondents (*N* = 161)

Characteristics	Intervention group	Control group	Total participants	Statistics
	*n*(%)	*n*(%)	*n*(%)	
Age				
Mean, SD	67.46(1.38)	63.52(1.43)	65.49(1.41)	*t* = 1.23 *p* = 0.21
Gender				
Male	34(42.0)	42(52.5)	76(47.2)	*χ* ^2^ = 1.78
Female	47(58.0)	38(47.5)	85(52.8)	*p* = 0.18
Race				
Malay	44(54.3)	40(50.0)	84(52.2)	*χ* ^2^ = 0.30
Non-Malay	37(45.7)	40(50.0)	77(47.8)	*p* = 0.58
Religion				
Muslim	44(54.3)	40(50.0)	84(52.2)	*χ* ^2^ = 0.30
Non-Muslim	37(45.7)	40(50.0)	77(47.8)	*p* = 0.58
Marital Status				
Single	8(9.8)	3(3.8)	11(6.8)	*χ* ^2^ = 3.28
Married	54(66.7)	62(77.5)	116(72.0)	*p* = 0.19
Others	19(23.5)	15(18.7)	34(21.1)	
Education level				
Illiterate	18(22.2)	22(27.5)	40(24.8)	*χ* ^2^ = 0.83
Diploma & less	47(58.0)	41(51.3)	88(54.7)	*p* = 0.65
Degree & above	16(19.8)	17(21.2)	33(20.5)	
Family history with cancer				
Yes	42(51.9)	32(40.0)	74(46.0)	*χ* ^2^ = 2.27
No	39(48.1)	48(60.0)	87(54.0)	*p* = 0.13
Cancer Stage				
Stage 1	7(8.6)	9(11.2)	16(9.9)	*χ* ^2^ = 1.03
Stage 2	16(19.8)	12(15.0)	28(17.4)	*p* = 0.79
Stage 3	30(37.0)	28(35.0)	58(36.1)	
Stage 4	28(34.6)	31(38.8)	59(36.6)	

*SD* standard deviation

### Change in the quality of life and each domain

Table 3 compares the mean scores for quality of life and each domain between the intervention and control groups at baseline until 3rd follow up. At baseline, there were no statistically significant differences between total mean score of quality of life (*t* = 0.54; *p* = 0.58), physical health (*t* = 0.63; *p* = 0.52), psychological health (*t* = −1.10; *p* = 0.27), social relationships (*t* = 1.63; *p* = 0.10) and environment (*t* = 0.64; *p* = 0.52) between the intervention and control groups. However, the mean differences of quality of life 81.95(95%CI 62.11–101.80; *p* = 0.000), physical health 21.01(95%CI 15.75–26.26; *p* = 0.000), psychological health 15.07 (95%CI 9.62–20.51; *p* = 0.000), social relationships 24.79(95%CI 18.92–30.67; *p* = 0.000) and environment 21.07(95%CI 15.86–26.29; *p* = 0.000) for the intervention group was significantly higher compared to the control group from baseline until 3rd follow up.

**Table 3:** Mean scores of quality of life and each domain between intervention and control group at baseline until 3rd follow ups

Quality of Life	Baseline	1^th^ follow-up	2^nd^ follow-up	3^rd^ follow-up	Effect of intervention	Statistics
		Mean ± SD			Mean differences (95% CI)	
Physical health						
Intervention Group	67.23 ± 21.40	70.25 ± 19.04	73.28 ± 17.08	76.17 ± 16.29	21.01, (15.75–26.26)	0.000
Control group	65.30 ± 16.69	54.06 ± 17.45	44.36 ± 16.42	39.18 ± 15.08	0	
Psychological health						
Intervention Group	60. 06 ± 19.55	60.68 ± 19.59	64.83 ± 18.84	67.09 ± 18.22	15.07, (9.62–20.51)	0.000
Control group	63.36 ± 18.39	51.37 ± 17.93	40.86 ± 16.83	36.78 ± 15.42	0	
Social relationships						
Intervention Group	63.35 ± 24.07	67.06 ± 21.40	71.62 ± 19.61	74.46 ± 18.43	24.79, (18.92–30.67)	0.000
Control group	57.76 ± 18.85	47.74 ± 18.70	37.83 ± 17.36	33.96 ± 16.39	0	
Environment						
Intervention Group	66.85 ± 20.99	70.57 ± 19.27	73.95 ± 17.42	76.40 ± 16.20	21.07, (15.86–26.29)	0.000
Control group	64.95 ± 16.41	54.63 ± 16.76	44.66 ± 16.26	39.21 ± 15.17	0	
Total quality of life						
Intervention Group	257.49 ± 80.00	268.55 ± 73.74	283.67 ± 67.92	294.11 ± 64.02	81.95, (62.11–101.80)	0.000
Control group	251.37 ± 60.79	207.80 ± 59.23	167.71 ± 56.85	149.12 ± 50.85	0	

The results of the two way repeated measures ANOVA analysis for each domain of QOL on both groups (intervention and control) and time (baseline until 3rd follow up) effects and interaction between group and time showed that; in physical health, there were significant main effects for group (*F* = 62.41, *p* = 0.001, partial Ƞ^2^ = 0.28), time (*F* = 78.92, *p* = 0.001, partial Ƞ^2^ = 0.33) and interaction between group and time (*F* = 311.56, *p* = 0.001, partial Ƞ^2^ = 0.66), in psychological health, there were significant main effects for group (*F* = 29.85, *p* = 0.001, partial Ƞ^2^ = 0.16), time (*F* = 101.42, *p* = 0.001, partial Ƞ^2^ = 0.39); and interaction between group and time (*F* = 295.19, *p* = 0.001, partial Ƞ^2^ = 0.65). Regarding social relationship, there was significant main effects for group (*F* = 69.58, *p* = 0.001, partial Ƞ^2^ = 0.30), time (*F* = 42.32, *p* = 0.001, partial Ƞ^2^ = 0.21) and interaction between group and time (*F* = 301.93, *p* = 0.001, partial Ƞ^2^ = 0.30). Finally in environment also, there was significant main effects for group (*F* = 63.74, *p* = 0.000, partial Ƞ^2^ = 0.29), time (*F* = 69.50, *p* = 0.001, partial Ƞ^2^ = 0.30) and interaction between group and time (*F* = 321.12, *p* = 0.001, partial Ƞ^2^ = 0.67).

### Change in the psychological effects (anxiety, depression)

Table 4 compares the mean scores for psychological effects (anxiety, depression) between the intervention and control groups at baseline, until 3rd follow up. At baseline, there were no significant differences of anxiety (*t* = −1.16; *p* = 0.24) and depression (*t* = 0.72; *p* = 0.47) between the intervention and control groups. However, the mean differences of anxiety −0.31, (−0.59–0.03; *p* = 0.028) and depression −0.56, (−0.85–0.27; *p* = 0.000) for the intervention group was significantly lower compared to the control group from baseline until 3rd follow up.

**Table 4:** Mean scores of psychological effects between intervention and control group at baseline until 3rd follow ups

Variables	Baseline	1^th^ follow-up	2^nd^ follow-up	3^rd^ follow-up	Effect of intervention	Statistics
		Mean ± SD			Mean differences (95% CI)	
Psychological effect Depression						
Intervention Group	1.40 ± 1.06	0.77 ± 0.83	0.56 ± 0.88	0.61 ± 0.87	−0.56, (−0.85–0.27)	0.000
Control group	1.28 ± 1.03	1.36 ± 1.00	1.43 ± 0.95	1.53 ± 1.00	0	
Anxiety						
Intervention Group	2.14 ± 0.80	1.80 ± 0.92	1.22 ± 1.02	1.20 ± 0.94	−0.31, (−0.59–0.03)	0.028
Control group	1.98 ± 0.93	1.90 ± 0.98	1.86 ± 1.00	1.88 ± 0.98	0	

The results of the two way repeated measures ANOVA analysis for anxiety and depression on both groups (intervention and control) and time (baseline, until 3rd follow-up) effects and interaction between group and time showed that; in depression, there were significant main effects for group (*F* = 14.93, *p* = 0.001, partial Ƞ^2^ = 0.09), time (*F* = 41.88, *p* = 0.001, partial Ƞ^2^ = 0.21)) and interaction between group and time (*F* = 103.40, *p* = 0.001, partial Ƞ^2^ = 0.40). In anxiety also, there were significant main effects for group (*F* = 4.95, *p* = 0.028, partial Ƞ^2^ = 0.03), time (*F* = 73.09, *p* = 0.001, partial Ƞ^2^ = 0.31); and interaction between group and time (*F* = 47.90, *p* = 0.001, partial Ƞ^2^ = 0.23).

### Changes in mean QOL and psychological scores within groups over time

To find out where the actual differences occurred pairwise comparison of baseline until 3rd follow up on QOL and physical effect scores was conducted. The differences in satisfaction scores were considered significant at *p* = 0.005 level (2-tailed) after Bonferroni adjustment. Regarding QOL, in all four domains of QOL (physical health, psychological health, social relationships and environment), the statistically significant differences were noted over time (*p* < 0.000). In psychological effects (anxiety, depression), the statistically significant differences for anxiety were noted over time (*p* < 0.000). Regarding depression, the statistically significant differences were noted over time (*p* < 0.000) except 1st to 2nd follow up (*p* = 0.11), 1st to 3rd follow up (*p* = 1.00).

## Discussion

### Summary of main finding

Both groups of control and intervention were matched in terms of socio-demographic and clinical factors at baseline (*p* > 0.05). There was a significant change between both groups in each domain of QOL (physical health, psychological health, social relationships, and environment) and psychological effect (anxiety and depression) scores from baseline until 3rd follow-up after each counselling session (*p* = 0.000). From the analysis, it is concluded that repetitive counselling by pharmacists was effective in improving each domain of QOL, and decreasing anxiety and depression for cancer patients.

### Comparison with existing literature

Quality of life is one of the most important concerns for cancer patients and survivors [25]. The results of the current study indicated that after counseling, the QOL of cancer patients in the intervention group significantly differed from the control group at all follow-ups. This significant difference verified the importance of repetitive counselling based on the “‘Managing Patients on Chemotherapy’” education module on improving QOL for cancer patients. In this regard, in western countries, many clinical practice guidelines have been developed for health care teams in psychotherapy and supportive care for improving QOL of cancer patients [26]. No significant changes were found in the QOL among the control group in this study. The findings of this study were found in line with studies done in India [27] and Iran [28] which reported that QOL of patients were improved after providing counseling for them. On the other hand, the significant difference in mean scores of psychosocial dimension of the QOL after consultation in the intervention group agrees with the results of the previous studies [29, 30]. Rehse and Pukrop [31] conducted a meta-analysis of 37 controlled studies and proves this issue, that counselling improve quality of life among cancer patients. Result of other meta-analysis study also supported the fact that education initiatives in the form of repetitive counselling by pharmacists can help in improving the cancer patient’s quality of life [30]. Detmar and colleagues [32] demonstrated that counselling by physicians made positive effects on cancer patients in regards to physiological health and physical health [32]. Also, Cappiello [33] believed that cancer patients experience many physical disorders during their treatment which can affect their QOL, and patients need support and counselling to cope with their condition.

#### Psychological effects (anxiety, depression)

Depression and anxiety are the most common psychological problems observed among oncology patients [34]. Cancer patients may experience anxiety or depression in different situations such as: waiting for the results, receiving a diagnosis, undergoing treatment, or anticipating a recurrence of their cancer [35]. Several studies showed that depression and anxiety are linked with higher mortality risk, worse pain control, poorer compliance and less desire for long-time therapy [34, 36]. There was significant improvement in this study with large effect size for depression (Ƞ^2^ = 0.40) and anxiety (Ƞ^2^ = 0.23) over time with repetitive counseling among cancer patients in the intervention group. This shows that repetitive counseling based on the “‘Managing Patients on Chemotherapy’” education module caused a reduction in depression and anxiety in the intervention group upon subsequent follow-ups. Similarly, result of a meta-analysis supported the present study as it clearly showed that psychosocial interventions with constant and frequent counseling revealed decreasing depression, anxiety and improving quality of life in adult cancer patients [30]. Results of other studies done in India [37] and China [38] supported the present study, where psychosocial interventions through counseling significantly reduced levels of depression and anxiety among cancer patients. Another study revealed that in addition to comprehensive counseling, the pharmacist played an important role in the treatment of depression through adherence and frequent follow-up [26].

#### Role of pharmacist

The management of cancer not only requires the prescription of the appropriate medicine but also require an intensive education and counseling of the patient [27]. The counseling helps in providing mental support and understanding about the disease, and also in improving the self-esteem of the patients [27]. Pharmacists, play a big role in improving the adherence to chemotherapy through effective counseling [39]. Our results highlighted the importance of counselling by pharmacists among cancer patients during their chemotherapy treatment for improving their quality of life and psychological effect(s). Trijsburg et al. [40] similarly reported that counseling had positive effect on mental and physical health which indirectly improves quality of life [40]. Besides, Mancini, et al. [39] reported that the goal of including a pharmacist in the care of patients with cancer is to avoid focusing only on oncology [39]. The study also stated that to treat patients with cancer, we must be able to treat the whole patient; this includes knowing, understanding, and reviewing the other co-morbidities these patients may have [39]. A pharmacist is uniquely trained to understand all the medications a patient may be using and how those interact with the cancer treatment medications [41].

#### Strengths and limitations

The major strength of this study includes the use of randomized control trial (RCT), single blind, and low attrition rate and suitable statistically tests. To the best of our knowledge, this is the first RCT study in Malaysia to develop chemotherapy counseling module on improving QOL among cancer patients receiving chemotherapy; consequently, the outcome of this study can be used as the fundamental data for further study. Besides that, using the validated Malay version of questionnaire for data collection increased the accuracy of the results, because patients were more fluent and comfortable in answering the questionnaire with in their national language. Along with the numerous strengths, this study has some limitation. First, all collected data were self-reported with no objective measures to evaluate the participants. The sample was recruited from one hospital which may affect the generalizability of the results so additional testing of our intervention is needed in other hospitals and different area in Malaysia.

#### Implication to practice

The module on ‘Managing Patients on Chemotherapy’ by pharmacists can be utilized for patients undergoing chemotherapy to minimize side effects of chemotherapy and improving QOL and decrease anxiety and depression among all oncology patients. Also, repetitive counseling conducted for every cycle would ensure its sustained effects among oncology patients undergoing chemotherapy.

## Conclusion

The current study demonstrates that the module of “Managing Patients on Chemotherapy” together with repetitive counseling by pharmacists was effective in improving QOL and decrease anxiety and depression of the oncology patients in the intervention group which will be result in the progress of quality of life in their families and the development of emotional, physical, and social activities in their families and society. Therefore, it is recommended that counseling by pharmacists should be included as part of the patients’ treatment program with the aim of reducing anxiety and depression and improving quality of life among them. On the otherhand, this module may be appropriate for future samples with similar demographic characteristics to improve QOL and psychological effects (anxiety, depression) of oncology patients in different hospitals in different area in Malaysia. The present study is the first study in Malaysia which has proven the effectiveness of repetitive chemotherapy counseling by pharmacists among oncology patients.

## Abbreviations

HRQOL: Health related quality of life
CDR: Cytotoxic drug reconstitution
FGD: Focus group discussion
GAD-7: Generalized anxiety disorder-7
GLM: Two-way repeated measures ANOVA
NCI: National Cancer Institute
NMRR: National Medical Research Register
PHQ-9: Patient health questionnaire-9
PTSD: Post-traumatic stress disorder
QOL: Quality of life
TAU: Treatment-as-usual
WHOQOL-BREF: WHO quality of life-BREF
